# *rac*-(1R*,2S*,3S*)-Diethyl 4-methyl-2-phenyl-6-(2-phenyl­hydrazinyl­idene)cyclo­hex-4-ene-1,3-dicarboxyl­ate

**DOI:** 10.1107/S1600536810045058

**Published:** 2010-12-18

**Authors:** Abel M. Maharramov, Arif I. Ismiyev, Bahruz A. Rashidov

**Affiliations:** a Baku State University, Z. Khalilov St. 23, Baku, AZ-1148, Azerbaijan

## Abstract

In the title compound, C_25_H_28_N_2_O_4_, the cyclohexene ring adopts a half-chair conformation and the dihedral angle between the aromatic rings is 59.44 (11)°. In the crystal, a weak intermolecular N—H⋯O hydrogen bond occurs.

## Related literature

For general background to Schiff bases, see: Cimerman *et al.* (1997 ▶); Offe *et al.* (1952 ▶); Richardson *et al.* (1988 ▶). For a related structure, see: Tamboura *et al.* (2009 ▶).
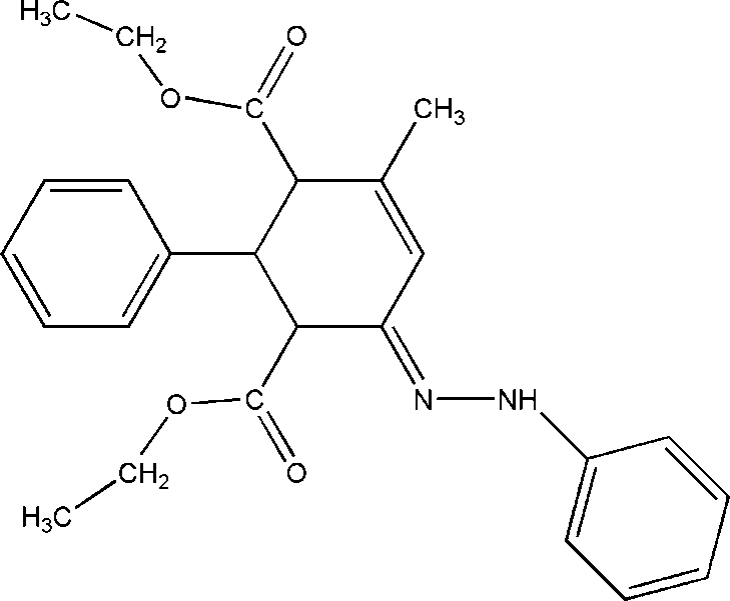


## Experimental

### 

#### Crystal data

C_25_H_28_N_2_O_4_
*M* *_r_* = 420.49
Monoclinic, 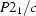

*a* = 11.5271 (10) Å
*b* = 13.4599 (12) Å
*c* = 14.4479 (13) Å
β = 93.342 (2)°
*V* = 2237.8 (3) Å^3^
*Z* = 4
Mo *K*α radiation
μ = 0.09 mm^−1^
*T* = 296 K
0.30 × 0.30 × 0.20 mm

#### Data collection

Bruker APEXII CCD diffractometer
Absorption correction: multi-scan (*SADABS*; Sheldrick, 1998 ▶) *T*_min_ = 0.975, *T*_max_ = 0.983
25227 measured reflections
5548 independent reflections
4052 reflections with *I* > 2σ(*I*)
*R*_int_ = 0.022

#### Refinement

*R*[*F* ^2^ > 2σ(*F* ^2^)] = 0.051
*wR*(*F* ^2^) = 0.149
*S* = 1.00
5548 reflections
289 parameters
H atoms treated by a mixture of independent and constrained refinement
Δρ_max_ = 0.34 e Å^−3^
Δρ_min_ = −0.24 e Å^−3^

### 

Data collection: *APEX2* (Bruker, 2005 ▶); cell refinement: *SAINT-Plus* (Bruker, 2001 ▶); data reduction: *SAINT-Plus*; program(s) used to solve structure: *SHELXTL* (Sheldrick, 2008 ▶); program(s) used to refine structure: *SHELXTL*; molecular graphics: *ORTEP-3 for Windows* (Farrugia, 1997 ▶); software used to prepare material for publication: *SHELXTL*.

## Supplementary Material

Crystal structure: contains datablocks global, I. DOI: 10.1107/S1600536810045058/zb2008sup1.cif

Structure factors: contains datablocks I. DOI: 10.1107/S1600536810045058/zb2008Isup2.hkl

Additional supplementary materials:  crystallographic information; 3D view; checkCIF report

## Figures and Tables

**Fig. 1.**
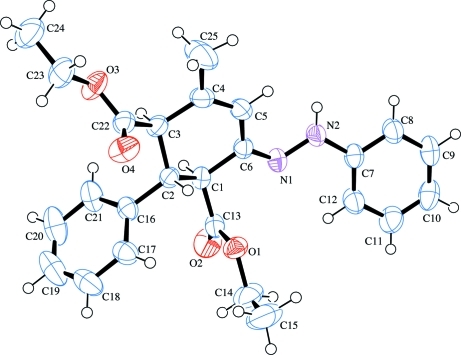
The molecular structure of the title compound, with the atomic numberingscheme. Displacement ellipsoids were drawn at the 50% probability level.

## supplementary crystallographic information

### Comment

Schiff bases have attracted much attention due to the possibility of their analytical applications (Cimerman *et al.*, 1997). They are also important ligands, which have been reported to have mild bacteriostatic activity and as potential oral iron-chelating drugs for genetic disorders such as thalassemia (Offe *et al.*, 1952; Richardson *et al.*, 1988). Metal complexes based on Schiff bases have received considerable attention because they can be utilized as model compounds of active centres in various complexes (Tamboura *et al.*, 2009). (rac)-Diethyl-4-methyl-2-phenyl-6-(2-phenylhydrazono)cyclohex-4-ene-1,3-dicarboxylate (I) have good antibacterial properties. We have synthesized the title compound, (I), and its structure is reported here (Fig. 1)..

### Experimental

(rac)-diethyl-4-hydroxy-4-methyl-6-oxo-2-phenyl-1,3-dicarboxylate (20 mmol), phenylhydrazine (20 mmol) were dissolved in 20 ml ethanol. The mixture was stirred at 345–350 K for 10 h. After cooling to room temperature white crystals were obtained. The crystals was filtered and washed with ethanol. recrystallization from ethanol (50 ml) yielded colourless block-shaped crystals of the title compound.

### Refinement

The hydrogen atoms of the NH-group (I) molecule were localized in the difference-Fourier map and included in the refinement with fixed positional and isotropic displacement parameters [*U*_iso_(H) = 1.5*U*_eq_(C) for CH_3_-group and *U*_iso_(H) = 1.2*U*_eq_(N) for amino groups]. The other hydrogen atoms were placed in calculated positions with and refined in the riding model with fixed isotropic displacement parameters [*U*_iso_(H) = 1.2*U*_eq_(C)].

### Crystal data

**Table:**

C_25_H_28_N_2_O_4_	*F*(000) = 896
*M**_r_* = 420.49	*D*_x_ = 1.248 Mg m^−^^3^
Monoclinic, *P*2_1_/*c*	Melting point: 444 K
Hall symbol: -P 2ybc	Mo *K*α radiation, λ = 0.71073 Å
*a* = 11.5271 (10) Å	Cell parameters from 8834 reflections
*b* = 13.4599 (12) Å	θ = 2.2–28.2°
*c* = 14.4479 (13) Å	µ = 0.09 mm^−^^1^
β = 93.342 (2)°	*T* = 296 K
*V* = 2237.8 (3) Å^3^	Block, colourless
*Z* = 4	0.30 × 0.30 × 0.20 mm

### Data collection

**Table:**

Bruker APEXII CCD diffractometer	5548 independent reflections
Radiation source: fine-focus sealed tube	4052 reflections with *I* > 2σ(*I*)
graphite	*R*_int_ = 0.022
φ and ω scans	θ_max_ = 28.3°, θ_min_ = 1.8°
Absorption correction: multi-scan (*SADABS*; Sheldrick, 1998)	*h* = −15→15
*T*_min_ = 0.975, *T*_max_ = 0.983	*k* = −17→17
25227 measured reflections	*l* = −19→19

### Refinement

**Table:**

Refinement on *F*^2^	Primary atom site location: structure-invariant direct methods
Least-squares matrix: full	Secondary atom site location: difference Fourier map
*R*[*F*^2^ > 2σ(*F*^2^)] = 0.051	Hydrogen site location: difference Fourier map
*wR*(*F*^2^) = 0.149	H atoms treated by a mixture of independent and constrained refinement
*S* = 1.00	*w* = 1/[σ^2^(*F*_o_^2^) + (0.0638*P*)^2^ + 0.859*P*] where *P* = (*F*_o_^2^ + 2*F*_c_^2^)/3
5548 reflections	(Δ/σ)_max_ = 0.004
289 parameters	Δρ_max_ = 0.34 e Å^−^^3^
0 restraints	Δρ_min_ = −0.24 e Å^−^^3^

### Special details

**Table:**

Geometry. All e.s.d.'s (except the e.s.d. in the dihedral angle between two l.s. planes) are estimated using the full covariance matrix. The cell e.s.d.'s are taken into account individually in the estimation of e.s.d.'s in distances, angles and torsion angles; correlations between e.s.d.'s in cell parameters are only used when they are defined by crystal symmetry. An approximate (isotropic) treatment of cell e.s.d.'s is used for estimating e.s.d.'s involving l.s. planes.
Refinement. Refinement of *F*^2^ against ALL reflections. The weighted *R*-factor *wR* and goodness of fit *S* are based on *F*^2^, conventional *R*-factors *R* are based on *F*, with *F* set to zero for negative *F*^2^. The threshold expression of *F*^2^ > σ(*F*^2^) is used only for calculating *R*-factors(gt) *etc*. and is not relevant to the choice of reflections for refinement. *R*-factors based on *F*^2^ are statistically about twice as large as those based on *F*, and *R*- factors based on ALL data will be even larger.

### Fractional atomic coordinates and isotropic or equivalent isotropic displacement parameters (Å^2^)

**Table:**

	*x*	*y*	*z*	*U*_iso_*/*U*_eq_	
O1	0.15913 (9)	0.34836 (9)	0.54219 (8)	0.0484 (3)	
O2	0.17111 (13)	0.50496 (11)	0.59085 (11)	0.0709 (4)	
N1	0.30804 (12)	0.45097 (10)	0.40168 (8)	0.0419 (3)	
C1	0.34611 (13)	0.41600 (11)	0.56061 (9)	0.0380 (3)	
H1B	0.3852	0.4757	0.5855	0.046*	
C2	0.38941 (12)	0.32697 (11)	0.61990 (9)	0.0361 (3)	
H2A	0.3507	0.2673	0.5944	0.043*	
C3	0.52040 (13)	0.31464 (12)	0.60926 (10)	0.0413 (3)	
H3A	0.5596	0.3754	0.6316	0.050*	
C4	0.54451 (14)	0.30086 (16)	0.50773 (12)	0.0542 (4)	
C5	0.47578 (14)	0.34400 (14)	0.44177 (11)	0.0490 (4)	
H5A	0.4929	0.3345	0.3803	0.059*	
C6	0.37661 (13)	0.40445 (11)	0.46046 (10)	0.0382 (3)	
C7	0.23724 (14)	0.47893 (11)	0.24792 (10)	0.0428 (3)	
N2	0.32965 (12)	0.45154 (10)	0.30993 (8)	0.0448 (3)	
H2B	0.3966	0.4359	0.2911	0.054*	
O3	0.67654 (10)	0.24545 (9)	0.69781 (9)	0.0594 (3)	
O4	0.51562 (11)	0.15407 (9)	0.68262 (10)	0.0594 (3)	
C8	0.25723 (17)	0.48808 (14)	0.15465 (11)	0.0531 (4)	
H8A	0.3309	0.4760	0.1341	0.064*	
C9	0.16733 (19)	0.51528 (16)	0.09237 (12)	0.0627 (5)	
H9A	0.1813	0.5221	0.0299	0.075*	
C10	0.05779 (19)	0.53242 (15)	0.12122 (13)	0.0646 (5)	
H10A	−0.0021	0.5506	0.0787	0.078*	
C11	0.03751 (18)	0.52248 (17)	0.21352 (14)	0.0658 (5)	
H11A	−0.0366	0.5339	0.2334	0.079*	
C12	0.12618 (16)	0.49575 (15)	0.27715 (12)	0.0565 (4)	
H12A	0.1115	0.4890	0.3395	0.068*	
C13	0.21677 (14)	0.43063 (12)	0.56620 (10)	0.0431 (3)	
C14	0.03416 (16)	0.34864 (19)	0.54926 (17)	0.0726 (6)	
H14A	−0.0022	0.3925	0.5029	0.087*	
H14B	0.0150	0.3720	0.6100	0.087*	
C15	−0.0078 (2)	0.2468 (3)	0.5345 (3)	0.0940 (9)	
H15A	−0.089 (3)	0.246 (3)	0.543 (2)	0.141*	
H15B	0.038 (3)	0.204 (3)	0.579 (2)	0.141*	
H15C	0.010 (3)	0.229 (3)	0.464 (3)	0.141*	
C16	0.35616 (14)	0.33795 (11)	0.71930 (10)	0.0418 (3)	
C17	0.26498 (15)	0.28237 (14)	0.75034 (12)	0.0519 (4)	
H17A	0.2261	0.2380	0.7101	0.062*	
C18	0.2309 (2)	0.29172 (18)	0.83973 (15)	0.0698 (6)	
H18A	0.1696	0.2537	0.8593	0.084*	
C19	0.2866 (3)	0.35647 (19)	0.89953 (15)	0.0816 (7)	
H19A	0.2641	0.3621	0.9601	0.098*	
C20	0.3760 (3)	0.41330 (18)	0.87026 (14)	0.0831 (7)	
H20A	0.4133	0.4583	0.9108	0.100*	
C21	0.4114 (2)	0.40414 (15)	0.78021 (12)	0.0633 (5)	
H21A	0.4724	0.4427	0.7609	0.076*	
C22	0.56773 (13)	0.22838 (12)	0.66713 (11)	0.0430 (3)	
C23	0.73944 (16)	0.16560 (15)	0.74630 (14)	0.0617 (5)	
H23A	0.7502	0.1102	0.7047	0.074*	
H23B	0.6968	0.1424	0.7980	0.074*	
C24	0.85377 (18)	0.2070 (2)	0.78001 (17)	0.0780 (7)	
H24A	0.8982	0.1564	0.8127	0.117*	
H24B	0.8418	0.2618	0.8209	0.117*	
H24C	0.8951	0.2295	0.7282	0.117*	
C25	0.6461 (2)	0.2380 (3)	0.48389 (17)	0.1185 (13)	
H25A	0.6504	0.2357	0.4178	0.178*	
H25B	0.6363	0.1719	0.5072	0.178*	
H25C	0.7164	0.2661	0.5115	0.178*	

### Atomic displacement parameters (Å^2^)

**Table:**

	*U*^11^	*U*^22^	*U*^33^	*U*^12^	*U*^13^	*U*^23^
O1	0.0358 (6)	0.0533 (7)	0.0561 (7)	0.0049 (5)	0.0021 (5)	−0.0080 (5)
O2	0.0700 (9)	0.0599 (8)	0.0818 (10)	0.0231 (7)	−0.0039 (7)	−0.0232 (7)
N1	0.0497 (7)	0.0413 (7)	0.0344 (6)	0.0034 (6)	−0.0012 (5)	0.0019 (5)
C1	0.0425 (8)	0.0365 (7)	0.0343 (7)	−0.0010 (6)	−0.0029 (6)	0.0003 (5)
C2	0.0357 (7)	0.0364 (7)	0.0360 (7)	−0.0020 (6)	0.0000 (5)	0.0017 (5)
C3	0.0348 (7)	0.0452 (8)	0.0433 (8)	−0.0026 (6)	−0.0031 (6)	0.0091 (6)
C4	0.0373 (8)	0.0774 (12)	0.0486 (9)	0.0092 (8)	0.0081 (7)	0.0155 (8)
C5	0.0427 (8)	0.0659 (11)	0.0389 (8)	0.0058 (7)	0.0065 (6)	0.0091 (7)
C6	0.0397 (7)	0.0394 (7)	0.0352 (7)	−0.0023 (6)	−0.0009 (6)	0.0024 (6)
C7	0.0527 (9)	0.0384 (7)	0.0367 (7)	0.0047 (6)	−0.0036 (6)	0.0012 (6)
N2	0.0461 (7)	0.0538 (8)	0.0342 (6)	0.0071 (6)	0.0004 (5)	0.0036 (5)
O3	0.0478 (7)	0.0564 (7)	0.0712 (8)	−0.0034 (5)	−0.0201 (6)	0.0201 (6)
O4	0.0492 (7)	0.0458 (7)	0.0826 (9)	−0.0026 (5)	0.0003 (6)	0.0151 (6)
C8	0.0588 (10)	0.0610 (10)	0.0392 (8)	0.0037 (8)	0.0005 (7)	0.0032 (7)
C9	0.0834 (14)	0.0677 (12)	0.0356 (8)	0.0030 (10)	−0.0093 (8)	0.0033 (8)
C10	0.0725 (13)	0.0640 (12)	0.0546 (11)	0.0151 (10)	−0.0213 (9)	−0.0022 (9)
C11	0.0568 (11)	0.0783 (13)	0.0610 (11)	0.0204 (10)	−0.0070 (9)	−0.0033 (10)
C12	0.0607 (11)	0.0679 (11)	0.0407 (8)	0.0168 (9)	0.0014 (7)	0.0012 (8)
C13	0.0485 (9)	0.0461 (8)	0.0343 (7)	0.0090 (7)	−0.0021 (6)	−0.0034 (6)
C14	0.0381 (9)	0.0907 (16)	0.0884 (15)	0.0118 (10)	−0.0004 (9)	−0.0160 (12)
C15	0.0479 (12)	0.112 (2)	0.122 (2)	−0.0180 (13)	0.0054 (13)	−0.0147 (18)
C16	0.0499 (9)	0.0390 (7)	0.0365 (7)	0.0062 (6)	0.0025 (6)	0.0059 (6)
C17	0.0531 (10)	0.0524 (9)	0.0511 (9)	0.0061 (8)	0.0100 (7)	0.0106 (7)
C18	0.0753 (13)	0.0769 (14)	0.0599 (12)	0.0185 (11)	0.0279 (10)	0.0203 (11)
C19	0.123 (2)	0.0778 (15)	0.0462 (11)	0.0353 (15)	0.0256 (12)	0.0136 (10)
C20	0.138 (2)	0.0670 (13)	0.0436 (10)	0.0085 (14)	−0.0038 (12)	−0.0099 (9)
C21	0.0915 (15)	0.0558 (11)	0.0421 (9)	−0.0110 (10)	−0.0010 (9)	−0.0008 (8)
C22	0.0402 (8)	0.0459 (8)	0.0427 (8)	−0.0002 (6)	−0.0003 (6)	0.0056 (6)
C23	0.0590 (11)	0.0592 (11)	0.0649 (11)	0.0090 (9)	−0.0131 (9)	0.0151 (9)
C24	0.0550 (12)	0.0930 (17)	0.0841 (15)	0.0032 (11)	−0.0133 (10)	0.0266 (13)
C25	0.0849 (17)	0.206 (4)	0.0673 (14)	0.085 (2)	0.0294 (13)	0.0412 (18)

### Geometric parameters (Å, °)

**Table:**

O1—C13	1.327 (2)	C10—H10A	0.9300
O1—C14	1.450 (2)	C11—C12	1.382 (3)
O2—C13	1.1944 (19)	C11—H11A	0.9300
N1—C6	1.2884 (19)	C12—H12A	0.9300
N1—N2	1.3630 (17)	C14—C15	1.465 (4)
C1—C13	1.511 (2)	C14—H14A	0.9700
C1—C6	1.5169 (19)	C14—H14B	0.9700
C1—C2	1.5392 (19)	C15—H15A	0.95 (4)
C1—H1B	0.9800	C15—H15B	0.99 (4)
C2—C16	1.515 (2)	C15—H15C	1.08 (4)
C2—C3	1.536 (2)	C16—C21	1.381 (2)
C2—H2A	0.9800	C16—C17	1.386 (2)
C3—C22	1.514 (2)	C17—C18	1.377 (3)
C3—C4	1.520 (2)	C17—H17A	0.9300
C3—H3A	0.9800	C18—C19	1.362 (4)
C4—C5	1.336 (2)	C18—H18A	0.9300
C4—C25	1.501 (3)	C19—C20	1.370 (4)
C5—C6	1.441 (2)	C19—H19A	0.9300
C5—H5A	0.9300	C20—C21	1.391 (3)
C7—C8	1.386 (2)	C20—H20A	0.9300
C7—C12	1.390 (2)	C21—H21A	0.9300
C7—N2	1.401 (2)	C23—C24	1.486 (3)
N2—H2B	0.8600	C23—H23A	0.9700
O3—C22	1.3257 (19)	C23—H23B	0.9700
O3—C23	1.453 (2)	C24—H24A	0.9600
O4—C22	1.1945 (19)	C24—H24B	0.9600
C8—C9	1.382 (3)	C24—H24C	0.9600
C8—H8A	0.9300	C25—H25A	0.9600
C9—C10	1.372 (3)	C25—H25B	0.9600
C9—H9A	0.9300	C25—H25C	0.9600
C10—C11	1.374 (3)		
			
C13—O1—C14	117.62 (14)	O1—C13—C1	110.96 (12)
C6—N1—N2	120.26 (13)	O1—C14—C15	107.96 (18)
C13—C1—C6	110.41 (11)	O1—C14—H14A	110.1
C13—C1—C2	111.15 (12)	C15—C14—H14A	110.1
C6—C1—C2	111.43 (12)	O1—C14—H14B	110.1
C13—C1—H1B	107.9	C15—C14—H14B	110.1
C6—C1—H1B	107.9	H14A—C14—H14B	108.4
C2—C1—H1B	107.9	C14—C15—H15A	108 (2)
C16—C2—C3	114.21 (12)	C14—C15—H15B	107 (2)
C16—C2—C1	111.09 (12)	H15A—C15—H15B	113 (3)
C3—C2—C1	108.50 (11)	C14—C15—H15C	106 (2)
C16—C2—H2A	107.6	H15A—C15—H15C	111 (3)
C3—C2—H2A	107.6	H15B—C15—H15C	111 (3)
C1—C2—H2A	107.6	C21—C16—C17	118.16 (16)
C22—C3—C4	111.04 (14)	C21—C16—C2	122.30 (15)
C22—C3—C2	110.64 (12)	C17—C16—C2	119.51 (14)
C4—C3—C2	110.24 (12)	C18—C17—C16	121.14 (19)
C22—C3—H3A	108.3	C18—C17—H17A	119.4
C4—C3—H3A	108.3	C16—C17—H17A	119.4
C2—C3—H3A	108.3	C19—C18—C17	120.3 (2)
C5—C4—C25	121.29 (17)	C19—C18—H18A	119.9
C5—C4—C3	120.07 (15)	C17—C18—H18A	119.9
C25—C4—C3	118.63 (15)	C18—C19—C20	119.80 (19)
C4—C5—C6	123.75 (15)	C18—C19—H19A	120.1
C4—C5—H5A	118.1	C20—C19—H19A	120.1
C6—C5—H5A	118.1	C19—C20—C21	120.4 (2)
N1—C6—C5	127.86 (14)	C19—C20—H20A	119.8
N1—C6—C1	114.30 (13)	C21—C20—H20A	119.8
C5—C6—C1	117.84 (12)	C16—C21—C20	120.3 (2)
C8—C7—C12	119.29 (15)	C16—C21—H21A	119.9
C8—C7—N2	118.82 (15)	C20—C21—H21A	119.9
C12—C7—N2	121.88 (14)	O4—C22—O3	123.97 (15)
N1—N2—C7	116.63 (13)	O4—C22—C3	125.21 (14)
N1—N2—H2B	121.7	O3—C22—C3	110.81 (13)
C7—N2—H2B	121.7	O3—C23—C24	106.77 (16)
C22—O3—C23	117.95 (14)	O3—C23—H23A	110.4
C9—C8—C7	119.73 (17)	C24—C23—H23A	110.4
C9—C8—H8A	120.1	O3—C23—H23B	110.4
C7—C8—H8A	120.1	C24—C23—H23B	110.4
C10—C9—C8	121.02 (17)	H23A—C23—H23B	108.6
C10—C9—H9A	119.5	C23—C24—H24A	109.5
C8—C9—H9A	119.5	C23—C24—H24B	109.5
C9—C10—C11	119.35 (17)	H24A—C24—H24B	109.5
C9—C10—H10A	120.3	C23—C24—H24C	109.5
C11—C10—H10A	120.3	H24A—C24—H24C	109.5
C10—C11—C12	120.66 (19)	H24B—C24—H24C	109.5
C10—C11—H11A	119.7	C4—C25—H25A	109.5
C12—C11—H11A	119.7	C4—C25—H25B	109.5
C11—C12—C7	119.93 (17)	H25A—C25—H25B	109.5
C11—C12—H12A	120.0	C4—C25—H25C	109.5
C7—C12—H12A	120.0	H25A—C25—H25C	109.5
O2—C13—O1	123.66 (16)	H25B—C25—H25C	109.5
O2—C13—C1	125.35 (16)		
			
C13—C1—C2—C16	−55.07 (15)	C10—C11—C12—C7	0.2 (3)
C6—C1—C2—C16	−178.68 (12)	C8—C7—C12—C11	−0.8 (3)
C13—C1—C2—C3	178.60 (12)	N2—C7—C12—C11	−179.88 (18)
C6—C1—C2—C3	54.99 (15)	C14—O1—C13—O2	−1.9 (2)
C16—C2—C3—C22	54.95 (17)	C14—O1—C13—C1	176.44 (15)
C1—C2—C3—C22	179.45 (12)	C6—C1—C13—O2	−113.05 (18)
C16—C2—C3—C4	178.17 (13)	C2—C1—C13—O2	122.76 (18)
C1—C2—C3—C4	−57.33 (16)	C6—C1—C13—O1	68.60 (16)
C22—C3—C4—C5	154.18 (17)	C2—C1—C13—O1	−55.59 (16)
C2—C3—C4—C5	31.2 (2)	C13—O1—C14—C15	−170.7 (2)
C22—C3—C4—C25	−25.6 (3)	C3—C2—C16—C21	49.1 (2)
C2—C3—C4—C25	−148.6 (2)	C1—C2—C16—C21	−74.00 (19)
C25—C4—C5—C6	179.5 (2)	C3—C2—C16—C17	−132.94 (15)
C3—C4—C5—C6	−0.3 (3)	C1—C2—C16—C17	103.95 (16)
N2—N1—C6—C5	−4.2 (2)	C21—C16—C17—C18	−0.8 (3)
N2—N1—C6—C1	176.16 (12)	C2—C16—C17—C18	−178.81 (16)
C4—C5—C6—N1	177.79 (18)	C16—C17—C18—C19	0.1 (3)
C4—C5—C6—C1	−2.6 (3)	C17—C18—C19—C20	0.7 (3)
C13—C1—C6—N1	29.81 (18)	C18—C19—C20—C21	−1.0 (4)
C2—C1—C6—N1	153.84 (13)	C17—C16—C21—C20	0.5 (3)
C13—C1—C6—C5	−149.86 (14)	C2—C16—C21—C20	178.51 (18)
C2—C1—C6—C5	−25.83 (18)	C19—C20—C21—C16	0.3 (3)
C6—N1—N2—C7	162.94 (14)	C23—O3—C22—O4	5.6 (3)
C8—C7—N2—N1	175.09 (14)	C23—O3—C22—C3	−173.54 (15)
C12—C7—N2—N1	−5.8 (2)	C4—C3—C22—O4	−89.5 (2)
C12—C7—C8—C9	1.1 (3)	C2—C3—C22—O4	33.2 (2)
N2—C7—C8—C9	−179.83 (17)	C4—C3—C22—O3	89.58 (17)
C7—C8—C9—C10	−0.7 (3)	C2—C3—C22—O3	−147.67 (14)
C8—C9—C10—C11	0.1 (3)	C22—O3—C23—C24	−175.08 (17)
C9—C10—C11—C12	0.2 (3)		
